# Contrast-enhanced T1-weighted image radiomics of brain metastases may predict *EGFR* mutation status in primary lung cancer

**DOI:** 10.1038/s41598-020-65470-7

**Published:** 2020-06-01

**Authors:** Sung Jun Ahn, Hyeokjin Kwon, Jin-Ju Yang, Mina Park, Yoon Jin Cha, Sang Hyun Suh, Jong-Min Lee

**Affiliations:** 1 Department of Radiology, Gangnam Severance Hospital, Yonsei University, College of Medicine, Seoul, Korea; 2 Department of Biomedical Engineering, Hanyang University, Seoul, Korea; 3 Department of Pathology, Gangnam Severance Hospital, Yonsei University, College of Medicine, Seoul, Korea

**Keywords:** Cancer imaging, Metastasis

## Abstract

Identification of *EGFR* mutations is critical to the treatment of primary lung cancer and brain metastases (BMs). Here, we explored whether radiomic features of contrast-enhanced T1-weighted images (T1WIs) of BMs predict *EGFR* mutation status in primary lung cancer cases. In total, 1209 features were extracted from the contrast-enhanced T1WIs of 61 patients with 210 measurable BMs. Feature selection and classification were optimized using several machine learning algorithms. Ten-fold cross-validation was applied to the T1WI BM dataset (189 BMs for training and 21 BMs for the test set). Area under receiver operating characteristic curves (AUC), accuracy, sensitivity, and specificity were calculated. Subgroup analyses were also performed according to metastasis size. For all measurable BMs, random forest (RF) classification with RF selection demonstrated the highest diagnostic performance for identifying *EGFR* mutation (AUC: 86.81). Support vector machine and AdaBoost were comparable to RF classification. Subgroup analyses revealed that small BMs had the highest AUC (89.09). The diagnostic performance for large BMs was lower than that for small BMs (the highest AUC: 78.22). Contrast-enhanced T1-weighted image radiomics of brain metastases predicted the *EGFR* mutation status of lung cancer BMs with good diagnostic performance. However, further study is necessary to apply this algorithm more widely and to larger BMs.

## Introduction

Lung cancer is one of the leading causes of cancer-related death worldwide, resulting in more than 1.18 million deaths annually^1–3^. Lung cancer commonly metastasizes to the brain, with 10–36% of all lung cancers developing brain metastasis (BM) during the course of the disease^4^. The incidence of BMs has increased in recent years, likely because of the prolonged survival of these patients. BM patients today undergo more efficient treatments and are assessed with better imaging techniques than were available previously, enabling the improved detection of BM^5,6^. Despite advanced therapies and improvements in survival rates, BM remains an important cause of morbidity associated with progressive neurologic deficits^7^.

Identification of the molecular subtypes of tumors using gene expression may allow a better understanding of their biology and patient-specific treatment: For instance, patients with gliomas with mutation of isocitrate dehydrogenase 1 gene (*IDH1*) or *IDH2* had better outcomes that those with wild-type *IDH* genes^8^. Also, O6-methylguanine DNA methyltransferase (MGMT) methylation status might be predictive of temozolomide (TMZ) response, a standard treatment for glioblastoma^9^. Breast cancer can be divided into three biologic subtypes, based on biomarkers, such as the estrogen receptor (ER), progesterone receptor (PR), and human epidermal growth receptor 2 (HER2); each subtype exhibits a distinct prognostic significance^10^. In the past several decades, identification of epidermal growth factor receptor (*EGFR*) mutations has become a critical part of treatment planning in advanced lung cancer and particularly in non-small cell lung cancer (NSCLC) cases^11^. Many recent studies have reported that patients with lung cancer and BMs harboring *EGFR* mutations exhibit improved survival over patients without the mutations due to higher response rates to whole-brain radiation therapy and specific chemotherapy medications. Such medications include *EGFR*-associated tyrosine kinase inhibitors (TKIs)^12–14^. *EGFR*-TKIs can be used as a first-line treatment for *EGFR* mutation-positive advanced NSCLC^15,16^.

Due to its relationship with differential treatment responses, the detection of *EGFR* mutation status with imaging biomarkers may improve clinical treatments and decision-making. A previous study found that BM imaging using a diffusion weighted approach in NSCLC cases allowed for good prediction of *EGFR* mutation status^17^. Recently, several studies have also used radiomics to extract primary brain tumor imaging features from contrast-enhanced T1-weighted images, a commonly used imaging modality^18–20^. However, the application of radiomic analyses of contrast-enhanced T1-weighted images to metastasis prediction has been rarely reported.

Radiomics is a growing field of diagnostic imaging that aims to non-invasively decode habitats by extracting large amounts of information on imaging features, by feature selection, and through data mining^21–23^. The heart of radiomics may be the extraction of high-dimensional features to capture attributes of habitats. Radiomic features can be divided into first-, second-, or higher-order statistical outputs. First-order outputs are generally based on histogram analyses and describe the distribution of values across individual voxels without concern for spatial relationships. Second-order outputs are generally based on texture analysis and describe statistical interrelationships between voxels with similar or dissimilar contrast values^21,24^. For instance, gray level co-occurrence matrix and gray level run length matrix are typical texture features^25,26^. Higher-order methods impose filters on medical images to extract repetitive or non-repetitive patterns^27–30^. For example, Laplacian transformations by Gaussian bandpass filtering can extract regions with increasingly coarse texture patterns^31^. Minkowski filters can assess patterns across voxels with an intensity above a given threshold^32^. Feature selection is used to resolve the “curse of dimensionality,” which refers to the problem that highly correlated and redundant features may cause overfitting and false discovery^33^. The most popular and readily-available feature selection algorithms include permutation random forest^34^, ℓ0-norm minimization^35^, infinite feature selection^36^, feature selection via concave minimization^37^, minimum redundancy maximum relevance^38^, relief^39^, and Laplacian^40^. Data mining is also a vital part of radiomics, which refers to the process of discovering patterns in large datasets. A range of machine learning algorithms have been introduced for data mining purposes, including random forest, support vector machine, adaptive boosting trees, and regularized logistic regression, which are widely used for learning and prediction^22,41^.

In the present study, we hypothesized that radiomics from contrast-enhanced T1-weighted images of BMs could be applied to predict *EGFR* mutation status in primary lung cancers. To test this, we extracted imaging features with first-, second, and higher-order methods and subsequently used different combinations of seven feature selection methods and four classification algorithms to identify the most robust analytic models.

## Materials and Methods

### Participants

We retrospectively reviewed data for a total of 146 lung cancer patients with BMs who underwent gadolinium-enhanced brain MRI at Gangnam Severance Hospital between June 2012 and July 2018. We excluded 85 patients for the following reasons: (1) previous neurosurgery or brain radiation therapy (n = 21), (2) presence of other malignant disease (n = 11), (3) poor image quality (n = 7), (4) absence of *EGFR* mutation status (n = 20), and (5) no measurable BM (n = 26). We regarded a BM as measurable when its diameter was greater than 3 mm, as it is difficult to differentiate BMs with a diameter of less than 3 mm from adjacent vessels. A total of 61 patients with 210 measurable BMs remained after exclusion. The institutional review board of Gangnam Severance Hospital approved this retrospective study and waived any requirement for informed consent because of its retrospective nature. All data were fully anonymized, and all experiments were carried out in accordance with approved guidelines.

### Pathology and EGFR mutation analysis

All patients had histopathological diagnoses of lung cancer by bronchoscopic, percutaneous needle-guided, or surgical biopsies. Genomic DNA was extracted from formalin-fixed, paraffin-embedded (FFPE) tissues using the DNeasy Isolation Kit (Qiagen, Valencia, CA, USA). We used the PNA Clamp^TM^ EGFR Mutation Detection Kit (PANAGENE, Daejeon, Korea) for detection of EGFR mutations by real-time PCR^42^.

### Image processing and extraction of radiomics features

**Figure 1:**
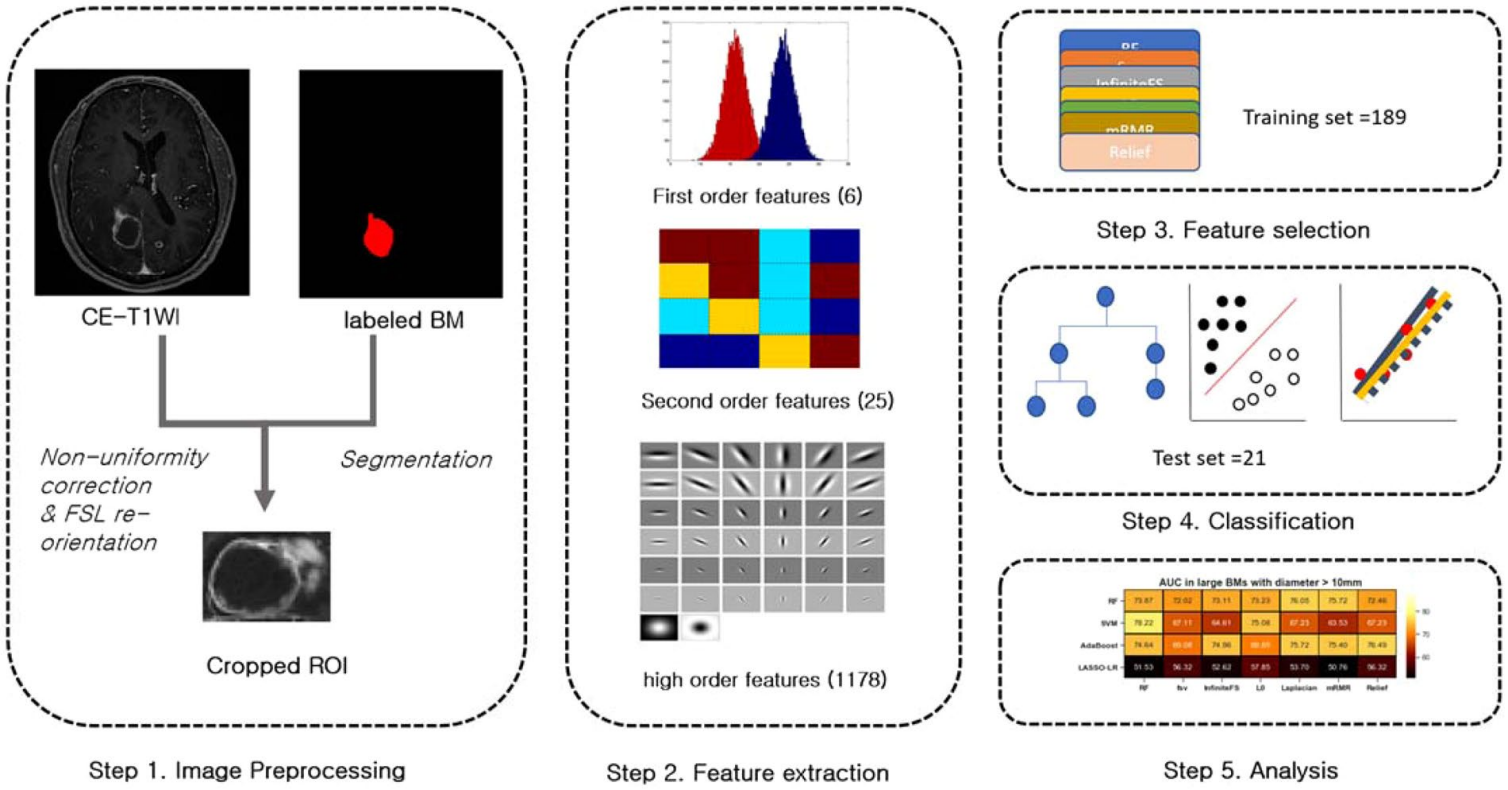
Flow diagram of the study design. (**a**) Segmentation was performed based on contrast-enhanced T1 weighted images (CE-T1WI). (**b**) 1209 features were extracted using first-, second- and higher-order methods. (**c**) Several combinations of seven selection methods and four classification algorithms were used. (**d**) Area under the curve, accuracy, sensitivity, and specificity were calculated.

T1-enhanced images were processed with the following steps: preprocessing, feature extraction, feature selection, and classification. For preprocessing, nonuniformity was corrected using the N3 bias correction algorithm, re-orientation was applied for further analysis using FMRIB Software Library (https://fsl.fmrib.ox.ac.uk/fsl/fslwiki), and cropped images including tumor volume were generated by a neuroradiologist (S.J.A) (Fig. 1). All imaging data were normalized to zero-mean and unit-variance to reduce bias. Radiomics features were extracted using MATLAB R2014b (MathWorks), in accordance with previous studies^18^. The 1209 resultant radiomics features comprised three feature groups: six first-order, 25 second-order, and 1178 higher-order features. First-order features were based on intensity profile histograms (e.g., for mean, variance, skewness, kurtosis, energy, and entropy, Supplemental Table 1). Second-order features were based on texture analysis consisting of 25 features^25,43,44^ (Supplemental Table 2). For higher-order features, 38 feature maps were created using the root filter set filter bank (Supplemental Table 3)^45,46^. Six first-order and 25 second order features were also generated for each feature map (1178 features).

### Feature selection and classification methods

A ten-fold validation method was applied to the data set (training set = 189, test set = 21). Feature selection was performed with a training set. A two-sample t-test of positive and negative classes was used for each feature to select the most discriminative features, to prevent overfitting, and to reduce feature space dimensions. Seven different feature selection algorithms were used for further feature selection: permutation random forest^34^, ${\mathscr{l}}0$-norm minimization^35^, infinite feature selection^36^, a feature selection via concave minimization^37^, minimum redundancy maximum relevance^38^, relief^39^, and Laplacian^40^.

Classification was performed with four different powerful algorithms to improve diagnostic performance for prediction of *EGFR* mutation: RF, support vector machine (SVM), adaptive boosting trees, and LASSO-regularized logistic regression^47–50^. These methods were chosen largely based on their common uses in previous studies and readily available implementation. Models were reestablished with features that were identified in the training set and then applied to the test set. Diagnostic performance was calculated using area under receiver operating characteristic curves (AUC), accuracy, sensitivity, and specificity. A subgroup analysis was performed depending on the size of the metastases (small vs. large). The diameter of small BMs was defined as less than 10 mm (n = 137) and that of large BMs was more than 10 mm (n = 73). For small BMs, ten-fold cross-validation was also used. However, for large BMs, the “leave one out method” was used to maintain a sufficiently large training dataset^51^.

### Statistical analysis

To evaluate a statistical significance of the classification performances, the permutation test was performed with a similar framework performed in previous studies^52,53^. We randomly permuted the group labels 500 times. In each permutation, the 10-fold cross-validation process was performed based on the permutated samples to calculate the AUCs. We defined p-value as follows;

P –value = (1 + number of time achieving higher AUCs than true lables) / 501(the number of all tests including the original one)

A threshold level of 0.05 was established for significance.

## Results

### Patient characteristics

Patient characteristics are summarized in Table 1. No significant differences were found in clinical characteristics between *EGFR*-wild type and *EGFR*-mutation groups. The mean ages at BM diagnosis were 64.0 ± 9.8 and 62.3 ± 11.6 years (*EGFR* wild type and *EGFR* mutation, respectively, p = 0.55). 65.6% of the *EGFR* wild-type patients (21/33) were male, and 51.7% of the *EGFR* mutation patients (15/29) were male (p = 0.35). Histologically diagnosed types of primary lung cancer included adenocarcinoma (27/32, 84.3% for *EGFR* wild type vs. 28/29, 96.6% for *EGFR* mutation) and small cell (5/32, 15.7% for *EGFR* wild type vs. 1/29, 3.4% for *EGFR* mutation, p = 0.26). In patients with EGFR mutation, 14 patients (48.3%) had exon 19 mutations, 11 patients (38%) had exon 21 mutations, 3 patients (10.3%) had exon 20 mutations, and one patient (3.4%) had a combined mutation of exon 19 and 20. Majority of BMs in our cohorts were diagnosed at initial screening (48/61, 79%) and there was no significant difference between two groups (24/32, 75% vs. 24/29, 82.7%, p = 0.67). The mean numbers of measurable BMs per patient were 3.5 ± 3.3 and 3.4 ± 3.0 mm (*EGFR* wild type and *EGFR* mutation, respectively, p = 0.90). The total number of measurable BMs was 210 (116 for *EGFR* wild type vs. 94 for *EGFR* mutation). The mean diameters of measurable BMs were 10.4 ± 7.4 and 10.8 ± 9.6 mm (*EGFR* wild type and *EGFR* mutation, respectively, p = 0.72). The total number of small BMs was 137 (75 for *EGFR* wild type and 62 for *EGFR* mutation). The mean diameters of measurable BMs were 5.8 ± 1.6 and 5.5 ± 1.7 mm (*EGFR* wild type and *EGFR* mutation, respectively, p = 0.31). The total number of large BMs was 73 (41 for *EGFR* wild type and 32 for *EGFR* mutation). The mean diameters of measurable BMs were 19.6 ± 6.4 and 22.2 ± 10.5 mm (*EGFR* wild type vs. *EGFR* mutation, respectively, p = 0.24).

**Table 1:** Lung cancer patient with brain metastases.

Characteristics	*EGFR* wild type (N = 32)	*EGFR* mutation (N = 29)	P-value
Age (years)	64.0 ± 9.8	62.3 ± 11.6	0.55
Sex			0.35
Male	21(65.6%)	15(51.7%)	
Female	11(34.4%)	14(48.3%)	
Histology			0.26
Adenocarcinoma	27(84.3%)	28(96.6%)	
Small cell	5(15.7%)	1(3.4%)	
**Subtype of EGFR mutation**			
Exon 18		0	
Exon 19		14 (48.3%)	
Exon 20		11 (38%)	
Exon 21		3 (10.3%)	
Exon 19&Exon 20		1 (3.4%)	
BM diagnosis at initial screening			0.67
Yes	24(75%)	24(82.7%)	
No	8(25%)	5(17.3%)	
Number of BMs per one patient	3.5 ± 3.3	3.4 ± 3.0	0.90
**All measurable BMs**			
Number	116	94	
Diameter (mm)	10.4 ± 7.4	10.8 ± 9.6	0.72
**Small BMs(Diameter ≤ 10 mm)**			
Number	75	62	
Diameter (mm)	5.8 ± 1.6	5.5 ± 1.7	0.31
**Large BMs(Diameter > 10 mm)**			
Number	41	32	
Diameter (mm)	19.6 ± 6.4	22.2 ± 10.5	0.24

brain metastases (BM); epidermal growth factor receptor (EGFR).

### Diagnostic performance

**Figure 2:**
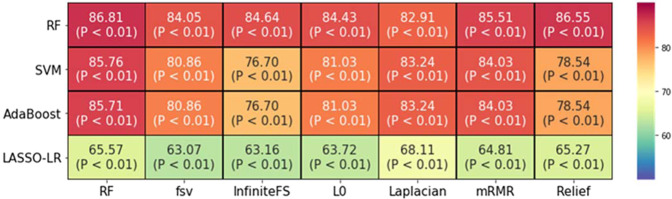
Heatmap of all brain metastases (BMs) depicting areas under the curve for seven feature selection (columns) and four classification (row) methods.

Using radiomic features, individual combinations of the seven selection features and four classification methods showed different *EGFR* diagnostic performances (AUC) for lung cancer BM (Fig. 2). The random forest classification using random forest selection demonstrated the highest AUC (86.81, p < 0.01). The sensitivity, specificity, and accuracy of this method were 84.41, 72.72, and 86.66, respectively. SVM and AdaBoost using the RF selection method also showed good diagnostic performances (AUC for SVM with RF: 85.76 and AUC for AdaBoost with RF: 85.71). However, LASSO-LR using Laplacian selection demonstrated a relatively poor diagnostic performance (AUC: 68.11, Table 2).

**Table 2:** Diagnostic performance of contrast-enhanced T1-weighted image radiomic-based prediction of *EGFR* mutation status in lung cancer brain metastases cases.

Classification	Best feature selection method	Optimal feature number	AUC	Sensitivity	Specificity	Accuracy
RF	RF	22	86.81	84.41	72.72	86.66
SVM	RF	17	85.76	82.07	81.81	86.19
AdaBoost	RF	18	85.71	83.093	72.72	85.23
LASSO-LR	Laplacian	48	68.11	55.03	81.81	69.04

Epidermal growth factor receptor (EGFR); area under the curve (AUC); random forest (RF); support vector machine (SVM).

### Subgroup analyses

**Figure 3:**
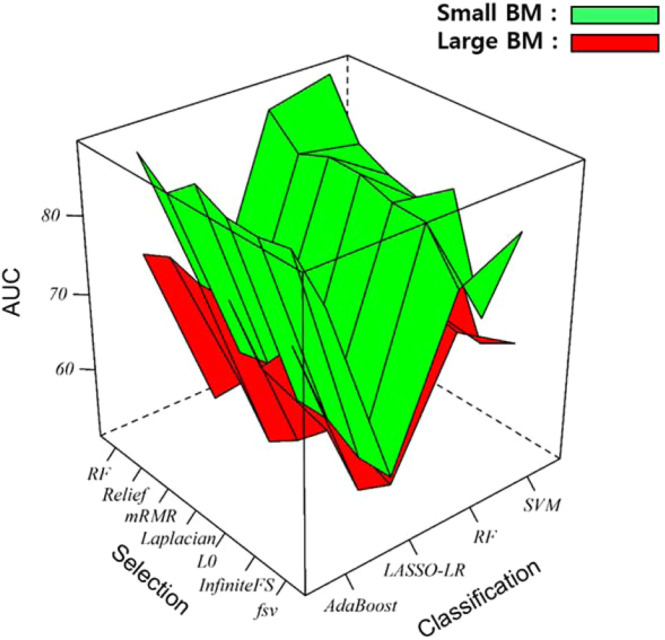
Multiple surface plots for (**a**) small brain metastases (BMs) (green) and (**b**) large BMs (red), depicting areas under the curve (AUC) for the seven feature selection (columns) and four classification (row) methods tested.

For small BMs, SVM classification using random forest selection demonstrated the highest AUC (89.09, Fig. 3a). The sensitivity, specificity, and accuracy of this method were 89.28, 100, and 89.06, respectively. AdaBoost with mRMR and RF with RF also had good diagnostic performances (AUC: 87.37 and 87.12, respectively). However, LASSO-LR using RF selection exhibited relatively poor diagnostic performance (AUC: 64.16, Table 3).

**Table 3:** Subgroup analysis of diagnostic performance for *EGFR* status in lung cancer brain metastases cases.

Subgroup	Classification	Best feature selection method	Optimal feature number	AUC	Sensitivity	Specificity	Accuracy
**Small BMs**							
	RF	RF	24	87.12	86.60	100	86.92
	SVM	RF	34	89.08	89.28	100	89.06
	AdaBoost	mRMR	35	87.37	88.21	100	86.92
	LASSO-LR	RF	26	64.16	65.17	71.42	63.51
**Large BMs**							
	RF	Laplacian	18	76.04	62.96	89.13	79.45
	SVM	RF	4	78.22	62.96	93.47	82.19
	AdaBoost	Relief	42	76.48	70.37	82.60	78.08
	LASSO-LR	L0	5	57.85	22.22	93.47	67.12

Brain metastases (BM), epidermal growth factor receptor (EGFR); area under the curve (AUC); random forest (RF); support vector machine (SVM).

For large BMs, SVM classification with RF selection demonstrated the highest AUC of 78.22 (Fig. 3b). The sensitivity, specificity, and accuracy of this method were 62.96, 93.47, and 82.19, respectively. AdaBoost with Relief and RF with Laplacian had similar diagnostic performances (AUC: 76.48 and 76.04, respectively). However, LASSO-LR with L0 demonstrated relatively poor diagnostic performance (AUC: 57.85, Table 3).

## Discussion

Tumor radiomics utilizes advanced computational methods to convert medical tumor images into a large number of quantitative features^54^. In the present study, we used seven feature selection methods and four classification methods to extract 1209 features from contrast-enhanced T1 images of 210 BMs. We analyzed the potential value of these features for predicting *EGFR* mutation status in primary lung cancer cases. We found that radiomics could be used to predict *EGFR* mutation status with high diagnostic validity. However, LASSO-LR demonstrated relatively poor diagnostic performance, compared with the other classification algorithms tested. Furthermore, diagnosing *EGFR* mutation status in large BMs (diameter > 10 mm) was not as effective as that in small BMs.

*EGFR* is a transmembrane protein with cytoplasmic kinase activity that transduces important growth factor signaling from the extracellular milieu into the cell^11^. Patients with lung cancer and BMs harboring *EGFR* mutations exhibit better responses to treatment as well as different clinical features. For example, the number of BM lesions was significantly higher in patients with *EGFR*-mutated NSCLC than in those with wild-type NSCLC. Moreover, leptomeningeal metastases were more common in patients with *EGFR*-mutated NSCLC^55^. A recent study proposed an imaging biomarker for the non-invasive determination of *EGFR* mutation status. Jung et. al reported that the minimum apparent diffusion coefficient (ADC) and normalized ADC ratio of BMs could be independent predictors of *EGFR* mutation status^17^. However, diffusion weighted images, which are used to calculate ADC variables, are not a routine sequence in BM protocols and parameters may thus vary between institutions. Meanwhile, contrast-enhanced T1 imaging is a common sequence in BM protocols because it is often used to delineate tumor margins and to monitor tumor responses to therapy. The clinical relevance of our results lies in the development of a novel imaging biomarker for BM *EGFR* mutation status in lung cancer patients. Of particular interest, this biomarker may be extracted from a commonly used sequence.

The high performance of *EGFR* mutation status prediction by our model can be explained by multiple factors. First, we generated first-, second-, and higher-order features using a root filter set filter bank. Higher-order features have been reported to help with capturing characteristic features: For example, one study found effective segmentation of white matter hyperintensities using a texton filter bank^56^. Furthermore, high-order CT features extracted through LoG and wavelet filters were used successfully to quantify non-small cell lung cancer phenotypes^21^. Second, we used a combination of several feature selection and data mining methods to achieve superior diagnostic performance.

Our results indicate that RF, AdaBoost, and SVM had good diagnostic performance, while LASSO did not. RF and AdaBoost are ensemble learning paradigms, which make predictions based on a number of different decision trees. However, their methodologies differ slightly. RF trains on multiple random subsets of features in a parallel way to arrive at a final conclusion^34^. Meanwhile, AdaBoost is trained on a number of decision trees sequentially, and each decision tree learns from mistakes made by the previous tree^57^. Generally, prediction variance decreases when the number of trees in the ensemble method increases. These models are insensitive to overfitting, which might explain their good performance^58^. SVM classifies by finding the hyperplane^59^. The hyperplane is calculated from the nearest training samples, called support vectors (SVs) and is optimized by maximizing the margin between the positive and negative SVs. As predicting *EGFR* status is a two-class problem (wild type or mutant), SV may be best suited for the purposes of the present study. LASSO is a variable selection algorithm used in regression models^50^. It adds a penalty equal to the absolute value of the magnitude coefficients. LASSO is a linear method and is preferred when true decision boundaries are linear. Thus, it appeared to struggle with handling nonlinear relationships in the data here. Given that LASSO had relatively poor performance in the present study, the relationship between the radiomics of contrast-enhanced T1WI of BMs and *EGFR* status is likely non-linear.

We identified RF as the most powerful selection tool of those tested here, regardless of classification method. RF selected related features based on importance scores, which are derived from how pure each feature is through numerous yes-or-no questions^34^. This process involves numerous decision trees, each of which is built via the random extraction of multiple features. Not every tree sees all of the features, guaranteeing that trees are de-correlated and therefore less prone to overfitting, a potential strength over other selection methods.

The performance of our model for large BMs was not as good as that for small BMs, which may be explained by several reasons. First, larger BMs tend to have necrotic centers that may affect machine learning classifications^17,60–62^. Critically, previous radiomics studies have used different ROI exclusion methods. For instance, Kickingereder et al. excluded ROIs with necrosis, while Kotrotsou et al. insisted that necrotic portions should be included in ROIs^63,64^. This issue should be further investigated in future work. Second, large BMs are associated with smaller datasets, potentially resulting in overfitting. However, cross-validation techniques and the random forest method diminishes the likelihood of such overfitting^34,65^.

Accumulating evidence suggests that there are clinico-pathological features that are closely related with EGFR mutations. Mutations have been shown to be associated with Asian ethnicity, adenocarcinoma histology, female sex, and non-smokers^11,66^. On the basis of results from a large study, these clinico-pathologic features of EGFR seem to be consistent in patients with lung cancer BMs^67^. In our results, the EGFR mutation group comprised more females and adenocarcinomas than the EGFR wild-type group, but the differences did not reach statistical significance. Thus, a combined model of clinico-pathologic features and radiomic model may enhance diagnostic performance for predicting EGFR mutation status in lung cancer BMs from larger populations which is expected to be validated in future study.

The present study has limitations that warrant consideration. Genetic testing was performed on lung samples rather than BMs themselves. Recent studies have revealed that *EGFR* mutation status in metastatic lesions does not always coincide with that at primary sites^55,68^. Indeed, discordant rates of *EGFR* mutation status between primary lung cancer and BM in previous studies range from 0 to 66.7%^69–75^. According to meta-analysis, the EGFR discordance rate between primary tumor and central nervous system is 17.26% (95% CI = 7.64 to 29.74)^76^. There are several models that might explain the discordance of *EGFR* mutation between primary lung cancer and BM. Cancer cells with highly diverse genetic profiles might be disseminated to distant organs at an early stage, or *EGFG* mutation status might change though multistep metastatic progression, potentially due to influences from the microenvironment and treatment effects. Thus, further study of tissues obtained directly from brain lesions or animal model with *EGFR* mutation is necessary to reveal the molecular and biologic characteristics of BMs more precisely. However, we believe our result has a clinical impact because it may aid in clinical decision for first-line treatment of lung cancer. The incidence of BMs in the patients with NSCLC at initial diagnosis is approximately 10%^4^. On the basis of this report, routine brain MRI screening scan is performed in many institution. Majority of BMs in our cohorts were also diagnosed at initial screening scan (48/61, 79%). In this perspective, our result may provide an alternative method to non-invasively assess EGFR information of primary lung cancer and offers a great supplement to biopsy, thereby making a proper first-line treatment of lung cancer. Also, our result is novel as it provides a different approach with previous other efforts using chest CT scan^77,78^.

In conclusion, we demonstrated here that T1-enhanced radiomics using RF classification may predict *EGFR* mutation status in lung cancer BMs with a high degree of accuracy. However, further study is necessary to apply T1-enhanced radiomics to large BMs.

## Supplementary information

Supplementary information.
