# Supplementary material for: Contrast-enhanced T1-weighted image radiomics of brain metastases may predict EGFR mutation status in primary lung cancer
Source: Sci Rep. 2020 Jun 1;10:8905. doi: 10.1038/s41598-020-65470-7 (PMC7264319; doi:10.1038/s41598-020-65470-7)
Supplement: Supplementary file 1 — Supplementary information. [file 41598_2020_65470_MOESM1_ESM.docx]

Supplemental Table 1. First order features

| number | category | feature |
| --- | --- | --- |
| 1 | 3d histogram | mean |
| 2 |  | variance |
| 3 |  | skewness |
| 4 |  | kertosis |
| 5 |  | energy |
| 6 |  | entropy |

Supplemental Table 2. Second order features

| number | category | feature |
| --- | --- | --- |
| 1 | 3D Gray level Run-length matrix (Rotation Invariant) | SRE |
| 2 |  | LRE |
| 3 |  | GLNUr |
| 4 |  | RLNU |
| 5 |  | RP |
| 6 |  | LGLRE |
| 7 |  | HGLRE |
| 8 |  | SRLGLE |
| 9 |  | SRHGLE |
| 10 |  | LRLGLE |
| 11 |  | LRHGLE |
| 12 | 3D Gray level Co-occurrence matrix (Rotation Invariant) | Angular second moment |
| 13 |  | GLCM Contrast |
| 14 |  | GLCM Correlation |
| 15 |  | GLCM Variance |
| 16 |  | Inverse Difference Moment |
| 17 |  | Sum average |
| 18 |  | Sum variance |
| 19 |  | Sum entropy |
| 20 |  | GLCM Entropy |
| 21 |  | Difference variance |
| 22 |  | Difference entropy |
| 23 |  | Information Measure of correlation 1 |
| 24 |  | Information Measure of correlation 2 |
| 25 |  | Maximal correlation coefficients |

SRE: short-run emphasis, LRE:long-run emphasis, LGLRE:low gray level run emphasis, HGLRE:high gray level run emphasis,GLNUr:gray level non uniformity, RLNU:run length non uniformity,SRLGLE:short run low gray level emphasis, SRHGLE:short run high gray level emphasis, LRLGLE:long run low gray level emphasis, LRHGLE: long run high gray level emphasis; RP: run percentage

Supplemental Table 3. 38 types of root filter set (RFS) bank

| number | filter | scale (𝜎_𝑥 ,𝜎_𝑦) | orientation |
| --- | --- | --- | --- |
| 1 | 1st derivatives of the Gaussian(bar filter) | (1,3) | 0 |
| 2 |  |  | 𝜋/6 |
| 3 |  |  | 2𝜋/6 |
| 4 |  |  | 3𝜋/6 |
| 5 |  |  | 4𝜋/6 |
| 6 |  |  | 5𝜋/6 |
| 7 |  | (2,6) | 0 |
| 8 |  |  | 𝜋/6 |
| 9 |  |  | 2𝜋/6 |
| 10 |  |  | 3𝜋/6 |
| 11 |  |  | 4𝜋/6 |
| 12 |  |  | 5𝜋/6 |
| 13 |  | (4,12) | 0 |
| 14 |  |  | 𝜋/6 |
| 15 |  |  | 2𝜋/6 |
| 16 |  |  | 3𝜋/6 |
| 17 |  |  | 4𝜋/6 |
| 18 |  |  | 5𝜋/6 |
| 19 | 2nd derivatives of the Gaussian(edge filter) | (1,3) | 0 |
| 20 |  |  | 𝜋/6 |
| 21 |  |  | 2𝜋/6 |
| 22 |  |  | 3𝜋/6 |
| 23 |  |  | 4𝜋/6 |
| 24 |  |  | 5𝜋/6 |
| 25 |  | (2,6) | 0 |
| 26 |  |  | 𝜋/6 |
| 27 |  |  | 2𝜋/6 |
| 28 |  |  | 3𝜋/6 |
| 29 |  |  | 4𝜋/6 |
| 30 |  |  | 5𝜋/6 |
| 31 |  | (4,12) | 0 |
| 32 |  |  | 𝜋/6 |
| 33 |  |  | 2𝜋/6 |
| 34 |  |  | 3𝜋/6 |
| 35 |  |  | 4𝜋/6 |
| 36 |  |  | 5𝜋/6 |
| 37 | Gaussian | σ=10 (rotational symmetry) | . |
| 38 | LoG | σ=10 (rotational symmetry) | . |
